# Surface Modification of PVDF Membranes for Treating Produced Waters by Direct Contact Membrane Distillation

**DOI:** 10.3390/ijerph16050685

**Published:** 2019-02-26

**Authors:** Mohanad Kamaz, Arijit Sengupta, Ashley Gutierrez, Yu-Hsuan Chiao, Ranil Wickramasinghe

**Affiliations:** 1 Ralph E Martin Department of Chemical Engineering, University of Arkansas, Fayetteville, AR 72703, USA; makamaz@email.uark.edu (M.K.); arijitbarc@gmail.com (A.S.); msdonaldc@hotmail.com (Y.-H.C.); 2 Department of Chemical Engineering, Prairie View A & M University, Prairie View, TX 77446, USA; ashleygutierrez33@yahoo.com

**Keywords:** fouling, hydraulic fracturing, polyionic liquid, surface modification, zwitterion

## Abstract

Direct contact membrane distillation (DCMD) has been conducted to treat hydraulic fracturing-produced water using polyvinylidenedifluoride (PVDF) membranes. Tailoring the surface properties of the membrane is critical in order to reduce the rate of adsorption of dissolved organic species as well as mineral salts. The PVDF membranes have been modified by grafting zwitterion and polyionic liquid-based polymer chains. In addition, surface oxidation of the PVDF membrane has been conducted using KMnO_4_ and NaOH. Surface modification conditions were chosen in order to minimize the decrease in contact angle. Thus, the membranes remain hydrophobic, essential for suppression of wetting. DCMD was conducted using the base PVDF membrane as well as modified membranes. In addition, DCMD was conducted on the base membrane using produced water (PW) that was pretreated by electrocoagulation to remove dissolved organic compounds. After DCMD all membranes were analyzed by scanning electron microscopy imaging as well as Energy-Dispersive X-Ray spectroscopy. Surface modification led to a greater volume of PW being treated by the membrane prior to drastic flux decline. The results indicate that tailoring the surface properties of the membrane enhances fouling resistance and could reduce pretreatment requirements.

## 1. Introduction

Increasing oil and gas production is predicted by the International Energy Agency with a corresponding increase in the volume of co-produced water that requires suitable treatment before disposal [1]. Here we focus on gas production by hydraulic fracturing operations. Oil and gas production from shale formations using hydraulic fracturing techniques has grown rapidly in the U.S. [2,3]. In order to extract oil and gas from these non-conventional reservoirs, water together with hydraulic fracturing fluids is injected at high pressure into the well in order to fracture the impervious rock formation that contains the trapped oil and gas. When the pressure is released flowback and co-produced water or ‘produced’ water (PW) is recovered. 

The PW is frequently highly impaired containing fracturing fluids as well as natural contaminants [4]. Treatment of PW is a major challenge [5,6]. Today deep well injection is frequently used to dispose the PW in the US in accordance with Environmental Protection Agency (EPA) regulations [7]. However cost and environmental concerns continue to be hurdles for implementation of this method of disposal. The simultaneous presence of large salt concentration in terms of total dissolved solids (TDS) and non-polar organic hydrocarbons makes the treatment of PW highly chemically challenging. 

Membrane technology using organic and inorganic membranes exhibits potential for treatment of oily-wastewater feeds having high TDS [8]. The low energy requirement of membrane-based technology is attractive [9]. Reverse osmosis (RO) has been used to treat PW with TDS in the range 500–25,000 mg/L. However, the high fouling potential of RO membranes means that adequate pretreatment of the PW is essential, increasing the overall processing costs [10]. Among other membrane-based processes, electrochemical-charge-driven separation processes including electrodialysis has been demonstrated as a technology for the treatment of PW. However, non-charged contaminants, including organic molecules, silica, and boron are poorly removed [11]. 

Membrane distillation (MD) is an emerging membrane based separation technology with high potential for treating different aqueous feed streams containing high TDS [12,13]. The vapor pressure difference across a porous hydrophobic gas filled membrane is the driving force for mass transfer across the membrane [14,15]. Water vapor as well as other volatile species will pass from the feed to the permeate side. However, passage of nonvolatile species and dissolved salts is suppressed. Since water vapor and not liquid water pass through the membrane pores the membrane must be sufficiently hydrophobic to suppress ‘wetting’ or passage of water together with dissolved salts and other nonvolatile species [16,17]. 

A major challenge for the commercial implementation of MD for treating PW is the presence of polar and nonpolar dissolved organic compounds that can easily adsorb onto the hydrophobic membrane surface. This can lead to membrane failure [18]. Deshmukh et al. [19] review many of the strategies used to modify the surface of the membrane in order to suppress fouling. In addition to flux decline, pore wetting is caused by adsorption of foulants such as surfactants and low surface tension dissolved species. 

Strategies to suppress fouling must, on a fundamental level, increase the energy barrier to foulant attachment as well as increase (i.e. make less negative) the Gibbs free energy for adsorption. Development of omniphobic membranes that exhibit high contact angles for water and non-polar organic compounds by tailoring membrane surface chemistry and morphology has been investigated [20]. However, while many of these approaches show promise, development of economically viable membrane casting and surface modification methods is likely to be challenging. 

Here we focus on surface modification of polyvinylidenedifluoride (PVDF) membranes which have been frequently used in membrane distillation studies [21]. Several methods of surface modification of PVDF membranes, including zwitterionic self-assembly, nanoparticle induced omniphobicity, inducing superhydrophobicity etc.; have been reported in literature in order to impart fouling resistance during membrane distillation [20,22,23,24]. In our previous work we have shown that electrocoagulation is effective in reducing the Total Organic Carbon (TOC) load in oily wastewater streams [18,25,26,27,28]. Specifically, for PW we have shown that membrane stability is significantly increased if the PW is pretreated using electrocoagulation. However, the viability of using electrocoagulation depends on the equipment as well as operating cost. Thus, by imparting greater resistance to fouling of the PVDF membrane by organic compounds a more robust membrane could be developed for which the pretreatment costs would be less. 

We have investigated two relatively simple surface modification method for altering the properties of the base PVDF membrane. Specifically, we aim to hydrophilize the surface by adding hydrophilic groups. While this could suppress adsorption of nonpolar organic compounds, it is essential the surface is resistant to wetting. Thus, we must maintain a high-water contact angle. In the first approach two different hydrophilic polymer chains have been investigated. Poly N-(3-sulfopropyl)-N-methacroyloxyethyl-N,N-dimethylammonium betaine (SAMB, zwitterionic polymer) has been grafted from the surface of the PVDF membrane. We have also grafted 1-allyl-3-vinylimidazolium bromide (Allyl, ionic liquid). In both cases the polymer chains were grafted using UV initiated free radical polymerization. The polymers contain fixed charges as well as hydrophobic segments. 

In the second approach we investigate the use of NaOH and KMnO_4_ to hydrophilize the membrane surface. Previous investigators [29,30,31,32,33] have indicated that incubating PVDF membranes in alkaline solution can lead to dehydrofluorination as fluoride in the PVDF backbone is replaced by hydroxide groups. In the case of KMnO_4_ Wang et al [34] have shown that incubating ploy(tetrafluoraethylene) (PTFE) films with KMnO_4_ in a nitric acid solution led to the replacement of fluoride by hydroxide and carbonyl groups. Here we use a similar procedure for PVDF membranes. In this second approach we avoid grafting an additional nanostructure from the membrane surface which could lead to an increase in the resistance to transport through the membrane and hence a decrease in permeate flux. 

Direct contact membrane distillation (DCMD) had been conducted using PW obtained from Southwestern Energy Fayetteville Shale (Arkansas, USA). The driving force for water vapor transport across the membrane is the vapor pressure difference generated by the temperature difference across the membrane. Base PVDF as well as modified PVDF membranes have been tested. In addition, the base PVDF membrane has been tested with water pretreated using electrocoagulation in order to compare results with the modified membranes. 

## 2. Materials and Methods

### 2.1. Materials 

Methanol, vinyl imidazole, allyl bromide, Poly N-(3-sulfopropyl)-N-(methacryloxyethyl)-N,N-dimethylammonium betaine (SAMB), potassium permanganate (KMnO_4_), and sodium hydroxide (NaOH) were purchased from Sigma Aldrich (St. Louis, MO, USA). Benzophenone was purchased from (Acros Organics, Morris, NJ, USA) while ethyl acetate was purchased from Alfa-Aesar (Ward Hill, MA, USA). Nitric acid (HNO_3_) was procured from VWR (Radnor, PA, USA). Deionized (DI) water was obtained from a Thermo Fisher 18 MΩ (Barnstead Smart2Pure system, Schwerte, Germany). Polyvinylidene fluoride (PVDF) membranes were provided by Millipore Sigma (Billerica, MA, USA). 

### 2.2. Characterization of Base Membrane

The characteristics of the PVDF membranes are summarized in Table 1.

### 2.3. Characterization of Produced Water (PW)

Hydraulic fracturing PW was used after pre-filtration using a screen filter to remove large particulate matter. The PW was characterized for total dissolved solid (TDS), total suspended solids (TSS), turbidity and total organic compounds (TOC) using EPA standard methods 160.1, 160.2, 415.1 and 180.1 [35], respectively as well as total nitrogen (TN) at the Arkansas Water Resources Center (Fayetteville, AR, USA). Cations and anions were measured using EPA methods 200.7 and 300.0, respectively, while metal ions were analyzed by Inductively Coupled Plasma Atomic Emission Spectrometry (ICP-AES). Table 2 summarizes the analytical results. The major ionic species present in the PW are found to be Ca, Na, Mg and Cl resulting in high TDS. The PW sample was also found to have a high TOC.

### 2.4. Synthesis of Ionic Liquid Monomer 

To prepare 1-allyl-3-vinylimidazolium bromide (Allyl) monomer, 0.025 M of vinyl imidazole and ally bromide were placed in a glass container and then the mixture was heated at 60 °C for three hours with vigorous stirring. Phase separation occurs after the reaction is complete with a viscous yellow color liquid setting to the bottom of the container. This is the ionic liquid phase. The top transparent layer was carefully discarded, and the ionic liquid phase was washed with excess ethyl acetate (three times) to remove unreacted precursors. The ionic liquid was used with no further purification. 

### 2.5. Membrane Modification

Prior to modification, the membranes were washed twice with (1:1) v:v water:ethanol to remove preservatives and impurities and then rinsed with DI water. Membrane coupons were cut (12.5 × 4.5 cm) and then modified.

#### 2.5.1. UV Grafting of Hydrophilic Polymers

The membranes were soaked in 50 mL methanol containing 15 g of benzophenone for 30 min. Since the membrane swells in methanol, benzophenone can enter the membrane matrix as well as adsorb on the membrane surface. The membranes were then air dried for 12 h at room temperature. Polymerization was conducted by placing the membrane coupon with active surface facing upwards in a petri dish. Next 5 mL of 10 mg/mL of the aqueous SAMB solution was added and the petri dish was placed in the UV reactor. In the case of the Allyl monomer, 2.4 mL of the monomer solution was added to 10 mL of DI water. The solution was then poured on top of the membrane and exposed to UV light. The UV reaction time was 5 min (UV irradiation 160 W). This time was chosen in order to ensure the resistance due to the grafted polymer chains did not lead to a significant decrease in permeate flux. Finally, the membranes were washed with DI water and dried at room temperature. 

#### 2.5.2. Surface Oxidation by KMnO4 and NaOH

KMnO_4_ (3 g) were dissolved in DI water (50 mL) and then nitric acid (3 mL) was added to the solution. The membrane was placed in a glass container containing KMnO_4_-HNO_3_ solution, with the active surface facing downwards. The container was then sealed securely and left for two hours at 60 °C. Next the membrane was taken out and washed with DI water thoroughly. For surface modification with NaOH a similar protocol was used. The membrane was incubated in 7.5 M NaOH at 70 ℃ for 30 min. 

### 2.6. Characterization of Modified Membranes

Fourier Transform Infrared (FTIR) Spectroscopy, Scanning Electron Microscopy (SEM), Energy-Dispersive X-Ray (EDX) Spectroscopy and water contact angle measurements were carried out in order to characterize the modified membrane surface. Each membrane sample was dried overnight prior to the analysis. The functional groups on the membranes were identified using FTIR spectroscopy. The FTIR spectra was recorded by an IR Affinity instrument (Shimadzu, Columbia, MD, USA) equipped with a PIKE single-reflection horizontal accessory. SEM images and EDX spectroscopy results were obtained using a Nova Nanolab 200 Duo-Beam Workstation (FEI, Hillsboro, OR, USA). The membrane surface hydrophobicity was measured using contact angle. The measurement was taken using a sessile drop contact angle goniometer (Model 100, Rame-Hart Instrument Company, Netcong, NJ, USA). Ten replicates were carried out for each measurement.

### 2.7. Direct Contact Membrane Distillation (DCMD) 

**Figure 1:**
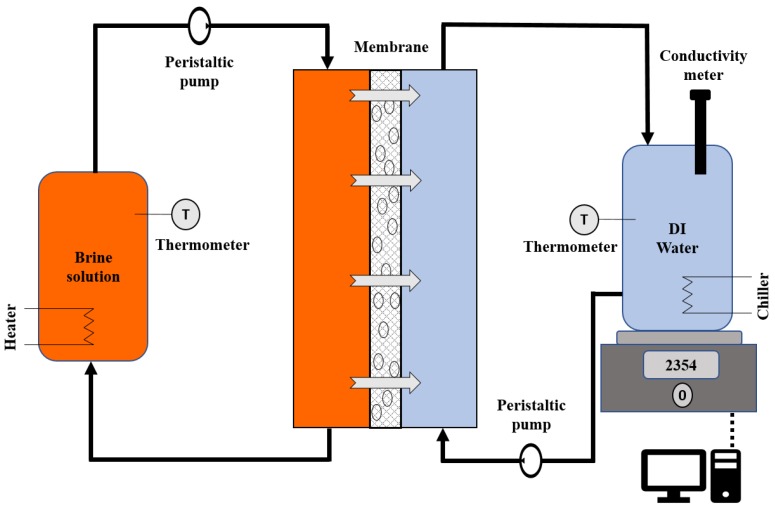
Schematic diagram of the DCMD apparatus.

In DCMD, the membrane is in direct contact with feed and permeate streams. The membrane was inserted between the two plates of the module and spacers stacked on top of the membrane from both sides to ensure an even flow distribution of the brine and DI water solutions over the membrane. The effective surface area of the membrane was 40 cm^2^. 1L of PW was used as feed while the temperature was kept at 60 ℃ by a heater. The permeate temperature was maintained at 10 ℃ using a chiller. The temperatures of the feed and permeate were controlled by heater and chiller procured from (PolyScience AD07R-40, Niles, IL, USA). A schematic diagram of the DCMD apparatus is given in Figure 1. The tank on the permeate side was placed on a computer-connected balance from (Mettler Toledo, Columbus, OH, USA) to measure the weight change every 5 min. The MD operation was performed at a fixed flow rate (0.45 L min^-1^) for both feed and permeate streams in countercurrent mode using two peristaltic pumps (Masterflex I/P, Cole Parmer, Vernon Hills, IL, USA). 

The permeate flux was determined by the equation below [36]:

(1) $$J=\frac{V_{p}}{A_{m}t}$$

where J is permeate flux expressed in Lm^-2^h^-1^. $V_{p}$ is the volume of water permeated in L, $A_{m}$ is the effective surface area of membrane expressed in m^2^, and t is the DCMD time in hour. The conductivity of the permeate was measured continuously during the operation by a conductivity-meter (VWR). If the permeate conductivity increased above 50 µS cm^-1^ it was assumed pore wetting had occurred which is associated with a rapid increase in the permeate flux. Prior to every DCMD run, both feed and permeate sides of the membrane were flushed with DI water at room temperature for one hour.

### 2.8. Pretreatment

Electrocoagulation was conducted to pretreat the feed [28]. Five electrodes (6061 aluminum alloy) with a surface area of 180 cm^2^ where inserted vertically in a 600 mL volume polycarbonate reactor with 5 mm spacing. A DC power source (Hewlett Packard, Palo Alto, CA, USA) was used with the cathode and anode attached to the first and last electrodes. 500 mL of the PW was transferred to the electrocoagulation reactor. The reaction was run for 30 s based on our earlier work. The current was kept constant at 0.5 A. The PW was then transferred to a separatory funnel for sludge sedimentation. After a 6 h sedimentation time, the clear supernatant was recovered and later tested using DCMD whilst the settled sludge was wasted.

## 3. Results and Discussion

### 3.1. Surface Modification

#### 3.1.1. Hydrophilic Polymer Grafting

Benzophenone, being a photo initiator, generated radicals on the PVDF surface. These radicals reacted with the oleophilic moieties of monomer through radical coupling reactions and hence the polymer chains grew. The grafting density is governed by the relative concentration of benzophenone and the duration of the initiator immobilization step. Longer immobilization times can immobilize more benzophenone, though a very high density of the polymer chains could lead to enhanced scale formation on the membrane surface. The duration of UV radiation will affect the length of hydrophilic polymers. The initiator concentration and UV irradiation time were chosen so that the grafted nanostructure would reduce fouling by organic compounds while minimizing any decrease in permeate flux due to the added resistance to permeate flow. 

#### 3.1.2. Surface Oxidation

**Figure 2:**
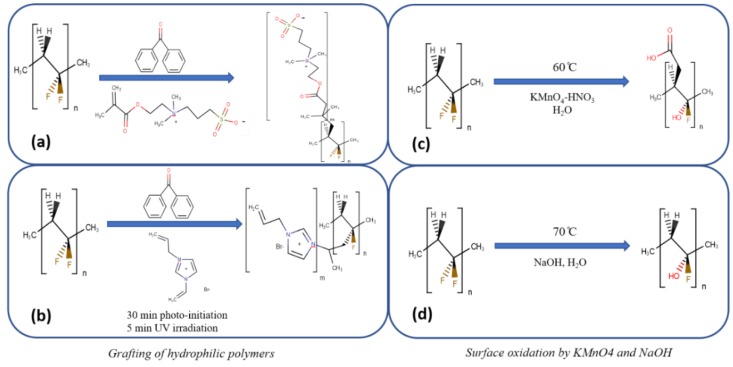
Schematic reaction schemes for modification of the PVDF membrane; (**a**) SAMB, (**b**) Allyl, (**c**) KMnO_4_, (**d**) NaOH.

Reaction conditions for dehydrofluorination were chosen in order to minimize damage to the membrane morphology while adding OH groups to the membrane surface. Figure 2 summarizes the membrane modification schemes.

### 3.2. Characterization

#### 3.2.1. FTIR Spectroscopy

**Figure 3:**
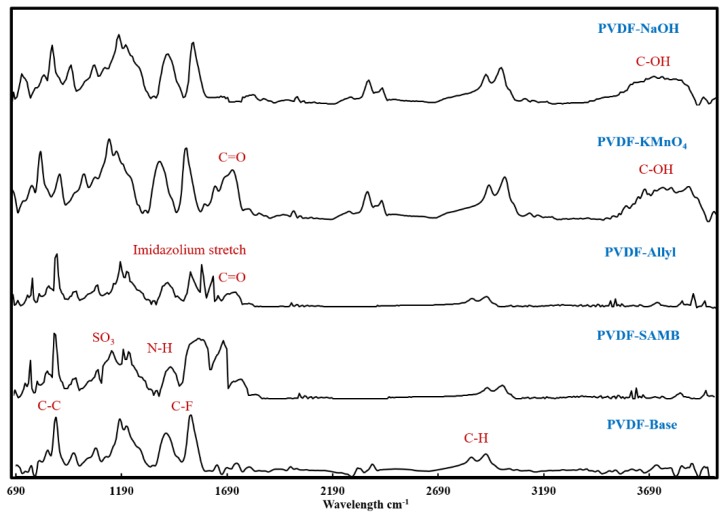
The FTIR spectra for virgin and surface modified PVDF membranes.

FTIR spectra for the base and modified membranes are given in Figure 3. For base PVDF membrane, the peaks ~ 860 cm^-1^, 1560 cm^-1^ and 2900 cm^-1^ were attributed to C-C, C-F and C-H bond stretching frequencies, respectively [20,21]. Grafting SAMB chains from the membrane surface was found to result in additional peaks in the FTIR spectra. The peaks ~1190 cm^-1^ and 1550 cm^-1^ were found to be signature peaks for –SO_3_^-^ and N-H moieties, respectively [37]. Similarly, grafting Allyl chains from the membrane surface resulted in signature peaks for the imidazolium ring at 1435 cm^-1^ and the carbonyl group around 1700 cm^-1^ [38]. Surface oxidation by KMnO_4_ resulted in highly intense –OH peak along with a carbonyl peak. Surface oxidation by NaOH showed a broad peak in the wavelength region 3300–3600 cm^-1^ that was attributed to –OH groups. The broadening of this peak is an indication of H bonding with other suitable moieties.

#### 3.2.2. Water Contact Angle Measurement

**Figure 4:**
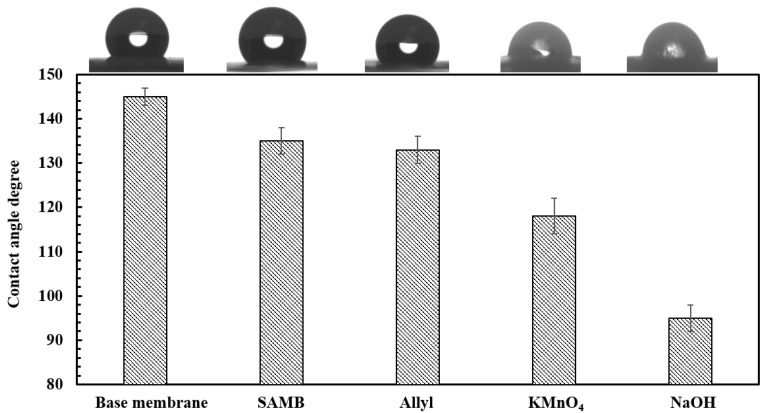
Water contact angles for base and modified membranes.

Surface water contact angles are given in Figure 4. 

As can be seen the base PVDF membrane is hydrophobic with a contact angle of 145° ± 2. Grafting SAMB and Allyl chains from the membrane surface was found to reduce the water contact angle slightly though the membranes are still hydrophobic. However, surface oxidation led to a much more pronounced decrease in contact angle. NaOH treatment led to the greatest decrease in contact angle. 

### 3.3. DCMD Performance

**Figure 5:**
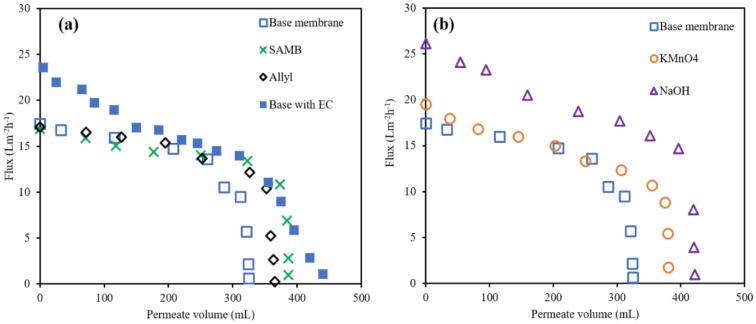
Variation of permeate flux as a function of permeate volume during DCMD, (**a**) gives results for the base and membranes modified by grafting polymer chains as well as the base membrane challenged with PW pretreated by electrocoagulation (EC); (**b**) gives results for the base membrane and membranes modified by surface oxidation.

Figure 5 gives the variation of permeate flux with permeate volume during DCMD. Figure 5a compares the flux results for the base membrane and SAMB and Allyl modified membranes as well as the base membrane challenged with PW pretreated by electrocoagulation. Figure 5b compares results for the base membrane and membranes modified via surface oxidation challenged with PW. In all cases the conductivity never rose above 50 µS cm^-1^. Thus, breakthrough of the feed was not observed. The initial flux for the base membrane was found to be 17 L m^-2^ h^-1^, which reduced gradually till 250 mL of permeate were removed followed a more drastic reduction. Though the flux declined and eventually stopped breakthrough was not observed. The productivity of the base membrane was about 300 mL of permeate. SAMB and Allyl grafting increased this value to be about 386 mL and 365 mL, respectively. The initial flux for the modified and base membrane is the same indicating little additional resistance to mass transfer through the membrane due to the grafted nanostructure.

**Figure 6:**
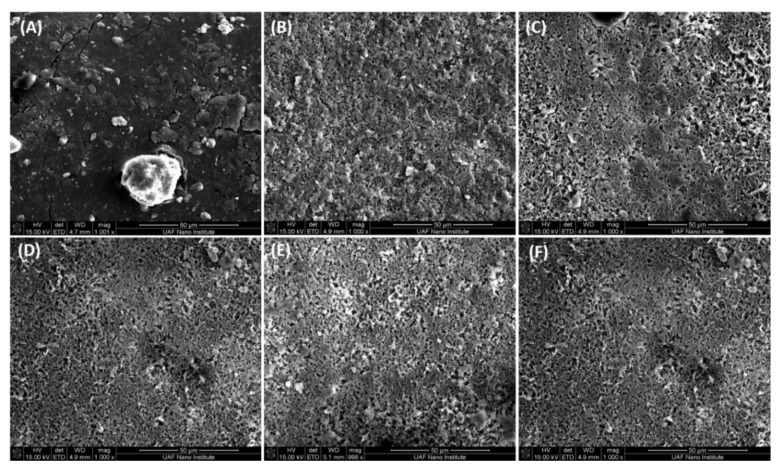
The SEM images for (**A**) base membrane after DCMD; (**B**) base membrane after DCMD with PW pretreated using EC; (**C**) SAMB modified membrane after DCMD; (**D**) Allyl modified membrane after DCMD; (**E**) KMnO_4_ treated membrane after DCMD; (**F**) NaOH treated membrane after DCMD.

Surface oxidation also led to an increase in productivity. As indicated in Figure 3, surface oxidation led to the presence of OH groups on the membrane surface. In the case of KMnO_4_ treatment, carbonyl groups are also present. NaOH treatment led to the greatest increase in productivity, greater than the three other surface treatments. The total permeate volume was around 420 mL. Figure 6 indicates that surface oxidation led to an increase in the initial water flux of the base membrane. This is not unexpected as surface oxidation not only imparts a more hydrophilic surface (see Figure 4) it also damages the membrane polymer. In fact, the degree of surface treatment must be carefully controlled as over treatment will lead to damage to the membrane. Results for the base membrane using PW pretreated by electrocoagulation indicate a slightly higher productivity of 440 mL as well as initial flux though there is a continuous flux decline. The result suggests that initially adsorption of organic species on the membrane surface occurs. In addition, when testing small membrane coupons there is much greater variation between the base membrane coupons which tend to even out in actual modules with large surface areas. 

### 3.4. Membrane Fouling 

The results in Figure 5 indicate that while no breakthrough occurs membrane fouling leads to a decrease in permeate flux. Membrane surfaces were analyzed after DCMD. Figure 6 gives SEM images for the base and modified membranes after DCMD using PW as well as the base membrane after DCMD with pretreated PW by electrocoagulation. In addition, SEM images are given of the modified membranes after DCMD. Finally, Table 3 gives the elemental analysis results from EDX spectroscopy.

Figure 5 indicates that adsorption on the membrane surface occurs for all membranes after DCMD though it appears more severe for the base membrane challenged with PW. This is in agreement with the rapid flux shown in Figure 5. Table 3 provides insights into the type of fouling that occurs. The base membrane contains C and F as is expected for PVDF. The presence of gold is due to the coating added prior to analysis. After DCMD the percentage of C and F on the surface decreases due to adsorption of rejected species from the PW. Comparing the elemental analysis for the base membrane after DCMD with PW and PW pretreated with electrocoagulation it can be seen that adsorption of inorganic species is higher for the pretreated PW. This is not unexpected as electrocoagulation is used to remove organic species. The result indicates that the base membrane is fouled by adsorption of both organic and inorganic species.

For the SAMB and Allyl modified membrane the percentage nitrogen is much higher than the KMnO_4_ and NaOH modified membranes. This is not unexpected as the grafted polymers contain N. In fact, it is similar to the percentage N detected for the base membrane after DCMD with PW. However, the amount of N for the KMnO_4_ and NaOH modified membranes is similar to that for the base membrane tested with pretreated PW by electrocoagulation. The pretreated PW will contain very little N associated with dissolved organic compounds. Taken together these results suggest that the modified membranes, especially modification by surface oxidization are more resistant to adsorption of organic compounds.

Again, all membranes indicate the presence of gold due to the coating used. Though there is some variation all modified membranes show higher amounts of adsorbed inorganic species on the surface than the base membrane after DCMD. For the base membrane challenged with pretreated PW, the amount of inorganic species detected on the surface is similar to the modified membranes. These results suggest that surface modification does not improve resistance to adsorption by inorganic species. It is important to note however, that the amount of water processed by the modified membranes especially the NaOH treated membrane is greater than the base membrane. 

Increasing the stability of the membrane during DCMD is critical to suppress wetting and flux decline during DCMD. For modified membranes, we have tried to minimize the decrease in the air/water contact angle. However, it is the underwater adsorption of solutes in the PW onto the membrane surface that is most relevant [39,40]. Air/water contact angles provide a general indication of the likely resistance of the surface to fouling. 

The results suggest that simple surface oxidation procedures could enhance the membrane resistance and increases membrane productivity. It may also lead to reduced pretreatment requirements. In the case of electrocoagulation which has been used here, optimizing reaction conditions will depend on the fouling resistance of the modified membrane as well as the quality of the PW. Optimization of the electrocoagulation conditions must minimize corrosion of the electrodes as well as power requirements. Further regeneration of the membrane after DCMD may be easier leading to longer membrane lifetimes. Here NaOH treated membranes showed the greatest improvement in performance. While this may be a simple and economical way to modify the base membrane, it is essential not to damage the membrane and degrade performance by over modification.

## 4. Conclusions

A major challenge for commercialization of DCMD is membrane stability due to the possibility of fouling by dissolved organic species as well as inorganic salts. Membrane fouling leads to flux decline and breakthrough of the feed into the permeate side. Four different surface modifications of base PVDF membranes have been investigated. Polymer chains consisting of zwitterionic groups as well as polyionic liquids and surface oxidation by KMnO_4_ and NaOH were studied. Modification conditions were chosen in order to minimize the decrease in water contact angle compared to the base membrane. 

All four modifications led to improved membrane productivity when tested with PW. For PW that was pretreated by electrocoagulation to remove dissolved organic compounds, the increases in productivity was the greatest. This result suggested that adsorption of dissolved organic compounds was a major cause of membrane fouling. Elemental analysis indicated that all modified membranes were more resistant to fouling by organic compounds though increased resistance to adsorption of inorganic species relative to the base membrane was not observed. The result suggests that simple surface modification procedures may enhance membrane fouling resistance thus improving membrane stability.

## Figures and Tables

**Table 1:** Characteristics of the PVDF membranes.

Membrane	Nominal Pore Size (μm)	Porosity (ε)	Thickness (δ) (μm)	d_mean_ Gas Permeation (μm)	Liquid Entry Pressure LEP (KPa)	Contact Angle	Tortuosity (τ)
PVDF	0.2	0.69	110	0.22	400	145° ± 2	2.01

**Table 2:** The characterization of Produced Water (PW).

Parameter	Concentration (ppm)
Calcium	24052
Magnesium	2463
Sodium	50379
Chloride	128786
Sulfate	8.6
TOC	181
TSS	1460
TN	15
TDS	202130
Turbidity	273 NTU

**Table 3:** EDX analysis for the virgin base membrane, base membrane after DCMD with PW pretreated by electrocoagulation as well as base and modified membranes after DCMD. EC = PW pretreated by electrocoagulation.

Element (at. %)	Base Membrane	After DCMD					
		Base	Base with EC	SAMB	Allyl	KMnO_4_	NaOH
C	41.3	32.7	35.4	40.3	38.9	37.7	35.8
F	56.1	23.4	24.7	19.1	18.4	13.3	12.1
O	-	10.4	3.1	4.6	2.6	19.8	21.4
N	-	5.2	1.9	4.8	5.9	1.4	0.8
Au	2.6	4.6	5.2	4.1	7.3	4.9	5.2
Na	-	6.3	7.4	6.8	7.3	7.1	8.1
Cl	-	6.9	10.5	8.5	8.7	6.7	7.6
Ca	-	8.4	7.4	9.3	8.1	5.6	6.4
Mg	-	2.1	4.4	2.5	2.8	3.5	2.6
